# Supplementary material for: Experimental and modeling study of the formation of cell aggregates with differential substrate adhesion
Source: PLoS One. 2020 Feb 5;15(2):e0222371. doi: 10.1371/journal.pone.0222371 (PMC7001941; doi:10.1371/journal.pone.0222371)
Supplement: S1 Appendix — (PDF) [file pone.0222371.s001.pdf]

Supplementary Material : Appendix

Image analysis with a FIJI macro

We combined pre-existing functions in FIJI in a home-made macro, in order to segment aggregates and measure their area, on each image taken every minute. Our algorithm works only with DIC images, with high contrast at the edges of aggregates. Those functions are:
“Find edges”(to detect the regions where the gradient of intensity is high, therefore in our images, contours of aggregates are detected); Gaussian blur (sigma = 1.00) (to smooth the aggregate contour and be sure to get a closed contour); “Make Binary” (in order to get a binary image with black pixels for the contours against white background), “Fill holes” (to fill holes inside cell contours). The area of the aggregates is measured by using the “Analyze Particles” function of FIJI.
In simulations, images are also produced. However, these images are binary so the “Analyze particle” function can be used directly.

Workflow

In our case we used a python script to organize, supervise and parallelize the following three main processes : the C++ implementation of our off lattice cellular automaton presented in details in section 2.5, the python script that produces images from the C++ simulation (mentioned in section 2.6) and the ImageJ image analysis macro. This supervising script launches simulations, produces images and launches image analysis.
The C++ program produces, for each timestep, a text file with the coordinate of each cells, their number of neighbors and the identification number of the aggregate they are part of. With the text files produced we can easily produce images with a very simple python routine. Finally, the ImageJ macro (for the simulations) described above is used to analyze the produced images.
